# Rootstock Breeding of Stone Fruits Under Modern Cultivation Regime: Current Status and Perspectives

**DOI:** 10.3390/plants14091320

**Published:** 2025-04-27

**Authors:** Juanjuan Ling, Wenjian Yu, Li Yang, Junhuan Zhang, Fengchao Jiang, Meiling Zhang, Yuzhu Wang, Haoyuan Sun

**Affiliations:** Institute of Forestry and Pomology, Beijing Academy of Agriculture and Forestry Sciences/Key Laboratory of Biology and Genetic Improvement of Horticultural Crops (North China), Ministry of Agriculture and Rural Affairs/Key Laboratory of Urban Agriculture (North China), Ministry of Agriculture and Rural Affairs/Beijing Engineering Research Center for Deciduous Fruit Trees/Apricot Engineering and Technology Research Center of the National Forestry and Grassland Administration, Beijing 100093, China

**Keywords:** stone fruits, rootstock, breeding objectives, breeding techniques, breeding achievements, strategies

## Abstract

Stone fruits (*Prunus* spp.) occupy a pivotal position in global fruit production due to their significant nutritional profile and distinctive organoleptic characteristics. Contemporary orchard systems are undergoing transformation through innovative cultivation approaches, notably high-density dwarfing systems, greenhouse cultivation, agri-tech integration, and simplified management. As a crucial agronomic component in modern stone fruit cultivation, rootstock systems confer multi-benefits including enhanced environmental resilience, improved scion productivity, superior fruit quality, controlled vigor, and dwarfing capacity. While the majority of European apple orchards have transitioned to dwarfing rootstock systems, achieving substantial gains in productivity and profitability, stone fruit cultivation lags significantly due to the key gaps in prunus rootstock development, including genetic complexity, extended evaluation cycles, clonal propagation barriers, and limited research programs. Urgent innovation is required to address these challenges in rootstock breeding to meet the demand of sustainable stone fruit production. This review systematically examines strategic breeding objectives and innovative molecular methodologies in prunus rootstock development, with particular emphasis on marker-assisted selection and genomic prediction technologies. We provide a comprehensive synthesis of breeding achievements across major commercial rootstock cultivars, while proposing forward-looking research strategies incorporating CRISPR-based genome editing and multi-omics approaches. The synthesized insights establish a theoretical pathway for advancing rootstock genetic improvement and sustainable orchard management practices in stone fruit cultivation systems.

## 1. Introduction

**Figure 1:**
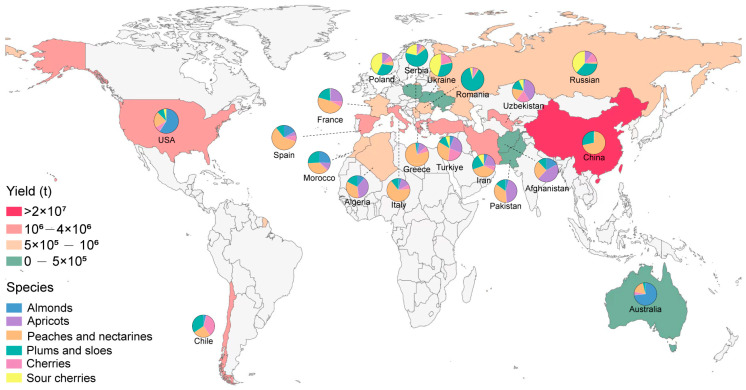
Production overview of the top 15 global stone fruit-producing countries in 2022.

Stone fruits, mainly encompassing sweet cherry (*Prunus avium*), sour cherry (*P. cerasus*), plums (*P. domestica* and *P. salicina*), peaches (*P. persica*), almonds (*P. dulcis*), and apricots (*P. armeniaca*) [1], constitute a vital horticultural group characterized by nutritional richness and organoleptic appeal. Beyond fresh consumption, their transformation into value-added products including jams, juices, dried fruits, and various desserts drives substantial global demand. In 2022, the global export value of stone fruit crops exceeded USD 10 billion, representing an approximate 47% increase compared to the previous decade [2]. Geospatial production patterns demonstrate a concentration within temperate zones between 30° and 40° latitude north latitude, where China maintains a dominant producer status, accounting for approximately 50% of the global total output (Figure 1) [2]. Due to strong economic returns, the global production of stone fruits has increased by 15% over the past decade. However, intensive land requirements and escalating labor inputs increasingly compromise grower profitability. In addition, the demand trend in the stone fruit market has shifted from pursuing high yield to focusing on quality. Therefore, this dual pressure necessitates the urgent implementation of sustainable intensification strategies through precision horticulture and genetic optimization to reconcile productivity with premium quality standards in stone fruit cultivation systems.

## 2. Modern Trends in Fruit Tree Cultivation Regime and the Demand for Advanced Rootstock Breeding

The global fruit production industry has undergone transformative changes in cultivation practices over the past century, driven by the need for higher productivity, resource efficiency, and labor optimization. Key trends in the modern fruit cultivation regime such as high-density dwarfing systems, protected cultivation, agri-tech integration, and simplified management protocols are reshaping orchard systems [3]. These models achieve efficient fruit cultivation and promote economic development by controlling tree height, regulating the environment, improving soil conditions, introducing mechanized operations, and building diverse ecosystems [4]. Among them, the implementation of dwarfing and dense planting not only directly reduces land use and facilitates manual harvesting but also makes it possible to construct greenhouses and introduce mechanized harvesting equipment [5]. It has become the mainstream model of modern fruit tree cultivation. The dwarfing and dense planting of apples is a successful case, with its core being the M series selected by the East Malling Research Station of dwarfing rootstocks and their derived varieties (such as the P series from Poland, the B series from Russia, and the G series from the United States) [6]. For example, rootstocks like ‘B.9’, ‘M.27’, and ‘M.65’ can reduce tree vigor by 50%, thereby significantly saving land resources and facilitating mechanized management and harvesting [7]. In addition, rootstocks can also eliminate or mitigate the limitations of various soil conditions, enhance tolerance to drought, waterlogging, salinity, and nutrient deficiency, provide resistance to viruses, bacteria, fungi, and pests, and improve yield and fruit quality [8]. Rootstocks have become an indispensable component in the modern cultivation mechanisms of fruit trees. While apple cultivation has achieved remarkable success through rootstock innovation, significant gaps persist in stone fruit rootstock development, reflecting both biological complexities and systemic research challenges. Fortunately, emerging biotechnologies and collaborative frameworks offer unprecedented opportunities to develop next-generation rootstocks. Prioritizing rootstock breeding is critical for climate-resilient, sustainable stone fruit production.

## 3. Breeding Objectives for Rootstocks of Stone Fruits

In fruit tree cultivation, an ideal rootstock should possess the ability to be easily propagated asexually, have strong environmental adaptability, exhibit strong compatibility with grafting, and effectively promote the dwarfing of the scion and enhance fruit quality. These are also the main breeding objectives for the rootstocks of stone fruit trees.

### 3.1. Efficient Clonal Propagation

Clonal rootstocks, due to their identical genotypes, exhibit minimal genetic variation among individuals, which allows for the maximum maintenance of uniform scion traits, thereby stabilizing yield and quality. In production, there are often potential rootstock varieties that possess multiple desirable traits but are unable to be propagated asexually at a large scale due to difficulties in adventitious root formation. Therefore, evaluating the adventitious root-forming ability of rootstocks is an important aspect of rootstock breeding. When breeding stone fruit rootstocks with ease of asexual reproduction as the primary selection trait, screening from a large pool of materials with rich genetic variation is a viable approach. Korkmaz et al. [9] compared the adventitious root formation ability of 79 plum rootstock cuttings, selecting 39 superior materials based on a threshold of root length of 33.50 mm, root number of 3, and rooting rate of 30%. Evaluating the rooting ability of rootstocks with other desirable traits is a method for further selection. A comparison regarding the adventitious root formation ability and quality of 30 rootstocks potentially tolerant to peach tree short-life syndrome (PTSL) showed that ‘GL-ERA-09-33’ (*P. persica*) had the best rooting efficiency, making it an excellent rootstock for tolerance to PTSL [10]. Among five *Prunus* rootstocks (‘Adafuel’ (*P. dulcis* × *P. persica*), ‘Adarcias’ (*P. dulcis* × *P. persica*), ‘Cadaman’ (*P. persica* × *P. davidiana*), ‘Garnem’ (*P. dulcis* × *P. persica*), and ‘GF677’ (*P. dulcis* × *P. persica*)), it was observed that the hardwood cuttings of ‘Garnem’ showed the best adventitious root formation ability, while ‘GF677’ and ‘Cadaman’ were less likely to root [11]. The difficulty in rooting ‘Cadaman’ is associated with a lower ratio of carbohydrates to nitrogen [12], while the difficulty in adventitious root formation for ‘GF677’ may be due to its higher activities of putrescine, polyamine oxidase, and catalase, as well as the lower activity of diamine oxidase [13].

### 3.2. Abiotic Stress Tolerance

Stone fruit trees exhibit a rich diversity of species and demonstrate remarkable adaptability to a wide range of environments. However, they often face various environmental stresses, including drought, waterlogging, soil salinization, and nutrient deficiencies. To mitigate these challenges, breeders have undertaken extensive screening efforts to select rootstocks with enhanced environmental adaptability. This review synthesizes current research progress in these key areas.

#### 3.2.1. Temperature Stress

Temperature is a crucial factor affecting the growth of stone fruits. Differences in temperature due to geographical latitude directly limit the ecological distribution of these fruits. Within suitable habitats, excessively high temperatures in summer or excessively low temperatures in winter can cause growth stagnation or even the death of these plants. Therefore, in the context of global climate change, the breeding of heat-tolerant and cold-tolerant rootstocks is of significant importance for maintaining the normal growth of stone fruits and expanding their cultivation areas. The cold tolerance of 48 cherry hybrid rootstocks was tested by Blážková et al. [14], using ‘Colt’ (*P. avium* × *P. pseudocerasus*) as a control, and they identified ‘CPHVODÁRNA’, ‘CPH 43’, ‘CPH 17’, ‘CPH 22’, and ‘CPH 49’ as new cold-tolerant rootstocks. Another study comparing the growth and physiological state of scions grafted onto cherry rootstocks ‘Gisela 5’ (*P. cerasus* × *P. canescens*) and ‘Mazzard’ (*P. avium*) under cold adaptation conditions showed that ‘Mazzard’ was better able to maintain fruit sugar metabolism and had a higher soluble sugar content, making it a more cold-tolerant cherry rootstock [15]. In addition, the response of seven *Prunus* rootstocks (‘Garnem’, ‘GF677’, ‘Marianna 2624’ (*P. cerasifera* × *P. musonianna*), ‘Myrobolan 29C’ (*P. cerasifera*), ‘Rootpac 20’ (*P. besseyi* × *P. cerasifera*), ‘Rootpac 40’ ((*P. amygdalus* × *P. persica*) × (*P. amygdalus* × *P. persica*)), and ‘Rootpac R’) to drought, heat shock, and their combination revealed that ‘Rootpac 40’ and ‘Marianna 2624’ consistently showed superior tolerance under stress, exhibiting a stem length increase of up to 30% and a 25% increase in stomatal conductance [16]. The most critical limiting factor for rootstocks is their adaptability to the local climate [17]. Compared with clonal rootstocks, the breeding of seedling rootstocks usually has distinct regional distribution characteristics. Therefore, specific seedling rootstocks often have stronger environmental adaptability. In the early stages, apricot cultivation also used ‘Haggith’ (*P. armeniaca*) seedling rootstocks to enhance cold hardiness [18].

#### 3.2.2. Water Stress

Water stress encompasses both drought and waterlogging, with the former preventing roots from absorbing adequate water and inhibiting various biological processes, while the latter causes oxygen deprivation in the roots, leading to the loss of normal biological functions. In cherries, a comparison of the root structure and anatomical characteristics of different genotypic rootstocks revealed that ‘SV2-7’ (*P. fruticosa*) and ‘OV14’ (*P. cerasus*) are drought-tolerant rootstocks [19]. Additionally, ‘Daqingye’ (*P. pseudocerasus*) and ‘Gisela 6’ (*P. cerasus* × *P. canescens*) were found to have higher levels of ROS scavenging ability and were more tolerant to waterlogging [20]. Except for clonal rootstocks, wild cherry (*P. microcarpa* and *P. incana*) seeding rootstocks can also be considered as a genetic source of drought resistance in cherry breeding programs [21]. In peaches and almonds, studies have shown that the wild almond (*P. ramonensis* and *P. webbii*) seeding rootstocks have greater tolerance to drought than commercial rootstocks, and are considered high-quality materials for breeding highly resistant rootstocks [22,23]. The latest report confirmed that *P. webbii* is one of the most drought-tolerant genotypes among four Prunus species (*P. webbii*, *P. elaeagnifolia*, ‘Garrigues’ (*P. dulcis*), and ‘Montclar’ (*P. persica*)) and ‘GF677’) under control, drought, and recovery conditions, based on a systematic analysis of enzymatic antioxidant systems, phytohormone profiles, and soluble sugar content under control, drought, and recovery conditions [24]. The rootstock ‘MP-29’ (*P. umbellata* × *P. persica*) is more flood-tolerant than the commonly used rootstock ‘Flordaguard’ (*P. persica*), making it suitable for promotion in subtropical regions prone to flooding [25]. Comparative studies of peach and plum rootstocks have found that among ‘Mr. S. 2/5’ (*P. cerasifera*), ‘Monegro’ (*P. persica* × *P. dulcis*), and ‘Nemared’ (*P. persica* × *P. davidiana*), the plum rootstock ‘Mr. S. 2/5’ has superior root porosity and greater tolerance to flooding [26]. Klumb et al. [27] also found that the plum rootstock ‘Marianna 2624’ has stronger waterlogging tolerance than the peach rootstock ‘NR0170401’ (*P. persica*) by assessing gas exchange parameters and changes in gene expression involved in glycolysis and ethylene metabolism under flooded conditions.

#### 3.2.3. Salinization

Soil salinization is another factor that affects the growth of root systems in stone fruit rootstocks. Over the past decade or so, there has been some success in the breeding of salt-tolerant rootstocks. A comprehensive salt tolerance analysis of seven *Prunus* rootstocks was conducted, identifying ‘Mariana 2624’, ‘Garnem’, and ‘Colt’ as having strong salt tolerance, with ‘Mariana 2624’ being recognized as the most salt-tolerant among the plum rootstocks [28]. Among peach and almond rootstocks, ‘Empyrean 1’ (*P. persica* × *P. davidiana*), ‘Cornerstone’ (*P. persica* × *P. dulcis*), and ‘Bright’s hybrid 5’ (*P. dulcis* × *P. persica*) also exhibit strong salt tolerance [29]. Furthermore, the high salt tolerance of the almond rootstock ‘Empyrean 1’ was further confirmed, with suggestions that its mechanism may involve upregulating ion transport proteins and enhancing the deposition of suberin and lignin in the root cortex to cope with salt stress [30].

#### 3.2.4. Nutritional Stress

The essential nutrients required by stone fruits include macronutrients such as N, P, K, and Ca, as well as micronutrients like Fe, Mn, and Zn, with both excess and deficiency of these nutrients leading to poor plant growth. Under nutrient-poor soil conditions, rootstocks with efficient nutrient uptake can help reduce the incidence of physiological diseases in the scion and decrease the amount of fertilizer needed. Reports show significant differences in the kinetic parameters of NO_3_^−^ and NH_4_^+^ absorption among different peach rootstocks, with varieties such as ‘Aldrighi’ (*P. persica*), ‘Tsukuba1’ (*P. persica*), and ‘Clone 15’ (*P. persica*) possessing a high-affinity transport system for NH_4_^+^, which facilitates nutrient translocation to the scion [31]. Similarly, the peach rootstock ‘Shannong-1’ exhibits higher nitrogen use efficiency compared to ‘Maotao’, a difference that is associated with the differential expression of nitrogen metabolism regulatory genes [32]. Additionally, the peach rootstock ‘Garnem’ may exhibit higher calcium use efficiency than the peach rootstock ‘GF677’ due to its larger cortical cells and secondary enlarged xylem vessels [33]. However, other reports suggest that ‘GF677’ has stronger tolerance to iron chlorosis, which may be attributed to the high expression of genes related to iron ion transport proteins, defense systems, and photosynthesis [34]. Additionally, using tolerant rootstocks to iron chlorosis is the best choice to prevent chlorosis. An evaluation of 17 *Prunus* rootstocks, conducted by measuring root ferric chelate reductase enzyme activity, leaf SPAD values, and field performance, identified ‘Adesoto’ (*P. insititia*), ‘Felinem’ (*P. dulcis* × *P. persica*), ‘GF677’, ‘Krymsk 86’ (*P. cerasifera* × *P. persica*), and ‘PAC 9921-07’ ((*P. besseyi* × *P. salicina*) × *P. armeniaca*) as having stronger tolerance to iron chlorosis [35]. In soils with excess elements, tolerant rootstocks are also a useful tool for improving scion growth. Studies have found that in soils with high zinc content, the cortical cells of the root systems of peach and plum rootstocks ‘Rigitano’ (*P. mume*) and ‘Tsukuba-1’ (*P. persica*) are prone to rupture, while ‘Flordaguard’ shows no significant changes in root tip morphology and anatomy, making it recommended as a rootstock with high zinc tolerance [36].

### 3.3. Biotic Stress Tolerance

In the production of stone fruit trees, pests and diseases not only directly cause poor growth but also lead to a decline in fruit quality and yield loss. The main pests and diseases affecting stone fruit trees include sharka, crown gall, root rot, and nematodes.

#### 3.3.1. Viral Diseases

*Plum pox virus* (PPV) is one of the most dangerous viruses affecting *Prunus* stone fruit trees, such as plum, apricot, peach, and cherry, causing host leaves to curl and lose their green color, leading to fruit deformities, altering fruit composition, and even resulting in premature fruit drop [37,38]. PPV typically spreads through infected propagation materials and aphid vectors, and has now been reported in at least 33 countries [38]. To select rootstocks resistant to PPV, Rubio et al. [39,40] evaluated the disease resistance of various stone fruit rootstocks and found that while ‘Myrotop’ (*P. cerasifera*), ‘Montclar’ (*P. persica*), ‘Citation’ (*P. salicina* × *P. persica*), ‘Evrica’ (*P. salicina* × *P. besseyi*), ‘ZH6’ (*P. persica* × *P. davidiana*), ‘GF677’ (*P. dulcis* × *P. persica*), and ‘L2’ (*P. lannesiana*) are considered resistant to PPV, ‘GF305’ (*P. persica*), ‘Puebla de Soto’ (*P. insititia*), and ‘Real Fino’ (*P. armeniaca*) are highly susceptible to PPV, ‘Myran’ ((*P. cerasifera* × *P. salicina*) × *P. persica*), ‘Viking’ (*P. persica* × (*P. dulcis* × (*P. davidiana* × *P. mume*))), ‘Julior’ (*P. insititia* × *P. domestica*), ‘Rubira’ (*P. persica*), ‘Myrobolan B’ (*P. cerasifera*), ‘MrS 2/5’ (*P. cerasifera* × *P. spinosa*), ‘Jaspi’ ((*P. salicina* × *P. cerasifera*) × *P. spinosa*), ‘MP8 ’(*P. domestica*), ‘Marianna 2624’ (*P. cerasifera* × *P. munsoniana*), ‘CP-2’ (*P. insititia*), and ‘AC 9921-07’ ((*P. besseyi* × *P. salicina*) × *P. armeniaca*) show susceptibility, and ‘Nemaguard’ (*P. davidiana* × *P. persica*), ‘Nemared’ (*P. persica* × *P. davidiana*), ‘STN2’ (*P. besseyi* × *P. cerasifera*), and ‘Torine’ (*P. domestica*) exhibit moderate susceptibility. In the breeding program in Germany, PPV-sensitivity rootstocks (‘Wavit’ (*P. domestica*) and ‘Weiwa’ (*P. domestica*)) as well as PPV hyper-sensitivity resistance rootstocks (‘Dospina 235’ (*P. domestica* × *P. spinosa*) and ‘Docera 6’ (*P. domestica* × *P. cerasifera*)) were created [41]. These results have opened up new possibilities for finding different sources of PPV resistance in Prunus stone fruit trees. Particularly, the grafting of ‘Garrigues’ (*P. dulcis*) induces PPV resistance in peach, and ‘Garrigues’ grafted as an interstock can serve as a kind of “vaccine” against PPV-Dideron in peach [42,43]. This resistance mechanism may be related to the induction of salicylic acid, but is distinct from systemic acquired resistance and induced systemic resistance necrosis responses [42]. The latest research suggests that ‘Garrigues’ may trigger a complex defense response in peach, thereby inducing PPV resistance, through the synergistic activation of antiviral defenses mediated by epigenetic modifications and small RNAs [44].

#### 3.3.2. Bacterial Diseases

Crown gall, caused by *Agrobacterium tumefaciens*, is one of the most susceptible bacterial diseases for stone fruit saplings such as apricot, peach, and almond [45]. An evaluation was conducted to screen for resistant rootstocks to mitigate the damage of this disease to stone fruit production, assessing the resistance of *Prunus* rootstocks ‘GF677’, ‘Antafuel’ (*P. amygdalus* × *P. persica*), ‘St.Julien GF 655/2’ (*P. insititia* × *P. domestica*), and ‘Gisela 5’, and the results showed that ‘St.Julien GF 655/2’ is insensitive to *A. tumefaciens* and exhibits the strongest resistance [46]. Bacterial canker, caused by *Pseudomonas syringae pv syringae* (Pss), is a serious disease of stone fruit orchards causing severe yield reductions and the death of entire trees. *Xylella fastidiosa* is known to infect many cultivated *Prunus* spp., such as almond, which is a very common host or even the main one, and peach, plum, apricot, and cherry, causing leaf scald, leaf scorch, and other symptoms such as twig dieback, stunted growth, and irregular leaf abscission [47,48,49]. New defense elicitor peptides, including BP100-fg15, HpaG23, FV7, RIJK2, PIP-1, Pep13, BP16-Pep13, fg15-BP100, and BP16, have been identified as suitable candidates to manage diseases caused by *X. fastidiosa*, particularly almond leaf scorch [50].

#### 3.3.3. Fungal Diseases

Root rot caused by *Armillaria mellea* or *A. tabescens* is a major cause of mortality in stone fruit trees, with research showing that plum rootstocks ‘Sharpe’ (*P. angustifolia* × *Prunus* spp.) and ‘MP-29’ exhibit resistance to *A. tabescens* [51,52], while ‘Myrobalan’ (*P. cerasifera*) and its interspecific hybrids demonstrate resistance to both *A. tabescens* and *A. mellea* [53]. Another significant root rot pathogen, *Phytophthora* spp., thrives in poorly drained soils, with modern intensive irrigation practices exacerbating waterlogging and root asphyxia issues that promote oomycete disease proliferation [54]. Studies on Prunus rootstock susceptibility reveal that ‘Nemaguard’ (*P. persica* × *P. davidiana*) resists *Ph. cambivora*, whereas ‘GF677’ is susceptible to *Ph. cactorum*, *Ph. citrophthora*, and *Ph. megasperma* [54]. Additional findings show that almond trees grafted onto ‘GF677’ are susceptible to *Ph. chlamydospora* [55], which also infects almond seedlings on ‘Nemaguard’ and ‘Lovell’ rootstocks [56], while hybrid rootstocks (‘Bright Hybrid-5’, ‘Bright Hybrid-106’, ‘Hansen-536’, ‘Garnem’) are vulnerable to *Ph. niederhauserii* [57].

#### 3.3.4. Pest Damage

Root-knot nematodes (RKNs), including *Meloidogyne hapla*, *M. arenaria*, *M. incognita*, and *M. javanica*, are among the most damaging pests to stone fruit trees. Since 1929, breeders have developed some peach rootstocks resistant to RKN, including ‘Nemaguard’, ‘Hansen’ (*P. persica* × *P. amygdalus*), ‘Flordaguard’, ‘Okinawa’, and ‘Nemared’ [58]. Eliwa et al. [59] evaluated that most genotypes of the local Egyptian peach variety ‘Mit-Ghamer’ (*P. persica*) exhibit resistance or moderate resistance to *M. incognita* and *M. javanica*. Unlike peach rootstocks, which are specifically resistant to certain types of RKN, the plum rootstock ‘Myrobalan’ has been found to be completely resistant to over 30 species of RKN, significantly mitigating the damage they cause to plum production [60]. Subsequent research further revealed that the broad-spectrum resistance of ‘Myrobalan’ is primarily attributed to three resistance genes: *Mal*, *Ma2*, and *Ma3* [61], which may activate the phenylpropanoid and flavonoid metabolic pathways to produce defensive secondary metabolites [62].

### 3.4. Graft Compatibility

The grafting compatibility between the scion and the rootstock is a crucial factor in determining the performance and longevity of an orchard. Generally, varieties and species with close genetic relationships exhibit good scion–rootstock compatibility, which is specifically manifested by the ability to establish effective vascular connections between the scion and rootstock [63]. Scion–rootstock incompatibility is typically divided into two scenarios: the first is “translocated” graft incompatibility, which manifests in the first year after grafting as growth cessation, leaf drop, and leaf discoloration; the second is “localized” incompatibility, which appears later in development as a disruption in the continuity of vascular and cambial patterns [64]. This also indicates that scion–rootstock incompatibility is delayed and unpredictable, often leading to significant economic losses. Therefore, it is essential to clearly understand scion–rootstock compatibility.

#### 3.4.1. Apricot

When selecting rootstocks for apricot scions, there is a strong universality in choice, as apricots have moderate compatibility with plum rootstocks (*P. cerasifera*, *P. salicina*, *P. mariana*, *P. insititia*, *P. domestica*). A comparison of the grafting compatibility of 13 apricot varieties with 4 plum rootstocks, ‘Marianna 2624’, ‘Miragreen’ (*P. cerasifera* × *P. davidiana*), ‘Mirared’ (*P. cerasifera* × (*P. persica* × *P. davidiana*)), and ‘Montclar’ (*P. persica*), found that over 90% of apricot varieties showed good compatibility with ‘Miragreen’ and ‘Mirared’, making them suitable as universal rootstocks for grafting apricot [65]. The Aula Dei Experimental Station observed through anatomical studies that apricot varieties (‘Búlida’, ‘Canino’, ‘Moniquí’, and ‘Paviot’) all exhibited good grafting compatibility with hexaploid plum rootstock (*P. insititia*) [66]. Moreover, apricot scions can also successfully graft onto peach, almond, or their hybrid seedlings [67]. Similarly, sand cherry (*P. pumila*, *P. besseyi*) and its interspecific hybrids are also suitable as rootstocks for apricots, but apricots should not be grafted onto Nanking cherry (*P. tomentosa*) [67].

#### 3.4.2. Plum

The selection of rootstocks for plum scions also exhibits considerable diversity. Myrobalan (*Prunus cerasifera*), the hybrid of Myrobalan and wild plum (*P. munsoniana*) known as ‘Marianna’, blackthorn (*P. spinosa*), European plum (*P. insititia*), and European plum (*P. domestica*) itself can all serve as rootstocks for European plum (*P. domestica*) [68]. Among them, Myrobalan and Marianna rootstocks are also commonly used for Japanese plum cultivars, especially in orchards with poor drainage where waterlogging is likely to occur. In some special cases, such as to avoid iron chlorosis in calcareous soils and to maintain good growth vigor of the scion in infertile soils, the interspecific hybrid of almond (*P. dulcis*) and peach (*P. persica*) is also selected as the rootstock for European and Japanese plums [64]. The Aula Dei Experimental Station tested the grafting compatibility of five European plum (*P. domestica*) varieties and six Japanese plum (*P. salicina*) varieties with 38 hybrid rootstocks, finding that Japanese plums ‘Angeleno’, ‘Black Amber’, ‘Delbarazur’, ‘Songold’, and European plums ‘President’ and ‘Reine Claude Tardive of Chambourcy’ showed perfect grafting compatibility with all tested rootstocks, while ‘Reine Claude Verte’ exhibited localized incompatibility with ‘Myrobalan B’ and ‘Myrobalan GF 3-1’ in the second year after budding, and ‘Stanley’ was only compatible with plum rootstocks [64].

#### 3.4.3. Peach

Traditionally, peach has often been grafted onto conspecific rootstocks (rootstocks of the same species), which are highly susceptible to diseases such as those caused by *Phytophthora* spp. To acquire resistance that is absent in conspecific rootstocks while maintaining high compatibility, breeders have developed hybrid rootstocks through crosses between peach and other stone fruit species within the Prunus genus [54]. Currently, *P. persica* × *P. dulcis* hybrids have been successfully developed as rootstocks for peach cultivars, including ‘Adafuel’, ‘Adarcias’, ‘Castore’, ‘Felinem’, ‘Garnem’, ‘GF677’, ‘IRTA-1’, ‘Pol-luce’, ‘Rootpac 40’, ‘Monegro’, and ‘Mayor’ [69,70]. In addition, studies have also identified a wide range of Prunus hybrid rootstocks suitable for peach cultivars, including two *P. davidiana* × *P. persica* hybrids (‘Barrier’and ‘Cadaman’), one *P. cerasifera* × *P. dulcis* hybrid (‘Replantpac’), three *P. salicina* × *P. persica* hybrids (‘Controller 5’, ‘Controller 9’, ‘PS’), and two composite hybrids (‘Ishtara’ (*P. cerasifera* × *P. salicina*) × (*P. cerasifera* × *P. persica*), ‘Rootpac 70’ (*P. persica* × *P. davidiana*) × (*P. dulcis* × *P. persica*)) [70,71,72].

#### 3.4.4. Almond

The rootstock of almond is similar to that of peach, and hybrid rootstocks are commonly used [73]. These include almond × peach hybrids such as ‘Garnem’ (*P. dulcis* × *P. persica*), ‘GF-677’ (*P. dulcis* × *P. persica*), ‘IRTA-1’ (*P. dulcis* × *P. persica*), and ‘Rootpac-40’ ((*P. dulcis* × *P. persica*) × (*P. dulcis* × *P. persica*)), as well as other Prunus hybrid rootstocks like ‘Rootpac20’ (*P. besseyi* × *P. cerasifera*), ‘IRTA-2’ (*P. cerasifera* × *P. dulcis*), ‘Replantpac’ (*P. cerasifera* × *P. dulcis*), and ‘Ishtara’ ((*P. cerasifera* × *P. salicina*) × (*P. cerasifera* × *P. persica*)) [69,73]. However, it is worth noting that the compatibility between scions and rootstocks is the result of an interaction. Montesinos et al. [74] found through transcriptome analysis of various almond grafting combinations that the scion ‘Lauranne’ (*P. amygdalus*) may exhibit better graft compatibility by promoting rootstock root development and nutrient absorption.

#### 3.4.5. Cherry

The selection of rootstocks for cherry scions is highly specific. For example, ‘Mazzard’ (*P. avium*), derived from sweet cherry seedlings, shows good grafting compatibility with all varieties of sweet cherry but exhibits incompatibility with rootstocks from different species, such as *P. mahaleb*, *P. cerasus*, *P. canescens*, *P. pseudocerasus*, *P. fruticosa*, and their hybrid progeny [75]. The accumulation of phenolic compounds and increased peroxidase activity may be the reasons for grafting incompatibility between cherry species or within the same species [76,77].

### 3.5. Dwarfing

Dwarfing rootstocks can control the growth vigor of fruit trees, effectively improve land use efficiency, increase the yield per unit area, and facilitate mechanized harvesting and orchard management, thereby reducing labor costs in production.

#### 3.5.1. Cherry

Sweet cherries are the first stone fruit trees to have commercial rootstocks with comprehensive vigor control, and the breeding effects of dwarfing rootstocks are the most systematic and significant. The semi-dwarfing rootstocks, including ‘Gisela 5’, ‘P-HL-C’ (*P. avium* × *P. cerasus*), and ‘Weiroot 158’ (*P. cerasus*), reduce tree height by about 50%; the full-dwarfing rootstocks, such as ‘Gisela 3’ (*P. cerasus* × *P. canescens*), ‘Lake’ (*P. avium* × *P. fruticosa*), ‘Clare’ (*P. avium* × (*P. cerasus* × *P. fruticosa*)), ‘Cass’ (*P. avium* × (*P. cerasus* × *P. fruticosa*)), ‘Crawford’ (*P. cerasus* × (*P. cerasus* × *P. canescens*)), and ‘Weiroot 53’ (*P. cerasus*), reduce tree height by about 60% [78].

#### 3.5.2. Peach and Almond

Up to now, breeders have also developed peach rootstocks with varying degrees of dwarfing. The ‘Sirio’ rootstock induces trees that are about 40% smaller than those of ‘GF677’ and can improve fruit yield and quality [58]. Compared with the standard rootstock ‘Nemaguard’, ‘Controller 5’ (*P. salicina* × *P. persica*) reduces tree vigor by 50–60%, while ‘Controller 9’ (*P. salicina* × *P. persica*) reduces it by about 90% [79]. Among ‘Rootpac 20’, ‘IRTA-1’, ‘Adesoto’, ‘Ishtara’, ‘Rootpac R’, and ‘Rootpac 40’, ‘Rootpac 20’ is the most dwarfing rootstock [80]. In addition, a peach dwarf mutant caused by a single nucleotide mutation in the gibberellin receptor GID1 has also been discovered, which has the potential to become an excellent dwarfing rootstock [81]. Recent reports, by comparing the growth physiological parameters of four almond cultivars—‘Lauranne’, ‘Tuono’, ‘Guara’, and ‘Belona’—grafted on two Prunus rootstocks, ‘Garnem’ and ‘Rootpac-40’, have found that the growth vigor induced by ‘Rootpac-40’ is relatively weaker, with the combination of ‘Rootpac-40’ and ‘Guara’ showing the lowest growth vigor [69]. Moreover, wild almond species *P. scoparia* seeding rootstock can be used as a dwarfing rootstock for almond [82].

#### 3.5.3. Plum and Apricot

The breeding of dwarfing rootstocks for plums and apricots has been underway for over 20 years, but progress in selection has been relatively slow, with preliminary evaluations of dwarfing rootstocks still ongoing in many regions. A recent study preliminarily selected 13 effective dwarfing rootstocks with strong rooting ability from 79 wild plum germplasms in the Euphrates River region [9]. A 16-year grafting experiment conducted at the Aula Dei experimental station found that the plum rootstock ‘Miral 3278 AD’ (*P. cerasifera × P. amygdalus*) significantly reduced the growth vigor of two European plum scions (‘Reine Claude of Bavay’ and ‘Reine Claude Tardive of Chambourcy’), while also promoting higher yields, making it a good alternative to the commercial rootstock ‘Myrobalan’ [83]. However, among the current major dwarfing rootstocks for plums and apricots, including ‘Krymsk 1’ (*P. tomentosa× P. cerasifera*), ‘St. Julien GF655/2’ (*P. insititia*), ‘Wavit’ (*P. domestica*), ‘Pixy’ (*P. insititia*), and ‘Pumiselect’ (*P. pumila*), most only achieve a semi-dwarfing effect [7,67]. In contrast, there are reports that two commonly used European plum rootstocks in Italy, ‘Penta’ (*P. domestica × P. cerasifera*) and ‘Tetra’ (*P. domestica*), can induce apricot trees to achieve a semi-dwarf to dwarf stature [67].

### 3.6. Others

Rootstock varieties have functionally differentiated effects on the yield and quality of drupes, with specific rootstocks potentially improving one or several quality indicators of the fruit. In peaches, the rootstock ‘KL-38’ (*P. cerasifera*) can increase the total phenol, total flavonoid, and total monomeric anthocyanin content to the highest levels, while the rootstocks ‘NG-1’ ((*P. davidiana* × *P. persica*) × (*P. dulcis* × *P. persica*)) and ‘NGF-14’ ((*P. davidiana* × *P. persica*) × *P. amygdalus*) can enhance the total antioxidant content [84].The rootstocks ‘Adarcias’ and ‘Cadaman’ can promote the accumulation of sugars, phenols, and flavonoids in peaches [85]. The peach rootstock ‘Rootpac 40’ can maintain good levels of soluble solids content and titratable acidity while also increasing fruit size [72].

However, research has found that the effects of rootstocks can be contradictory between fruit yield and quality, and even among different quality indicators. For example, ‘GF677’ significantly improves iron chlorosis tolerance but results in the smallest fruit size, while the rootstocks ‘Barrier1’ (*P. persica* × *P. davidiana*) and ‘Citation’ (*P. persica* × *P. salicina*) can significantly increase the acidity of fruit but reduce its nutritional value [86]. The peach–plum hybrid rootstock ‘PS’ (*P. persica* × *P. cerasifera*) can increase the sugar, total phenol, and ascorbic acid content in nectarines, enhancing their antioxidant activity, but it is sensitive to iron chlorosis [72,87]. Similarly, the sweet cherry rootstock ‘Pikú 3’ (*P. pseudocerasus* × (*P. canescens* × *P. incisa*)) may result in lower yields for the ‘Newstar’ variety, but it produces larger fruits with higher soluble solids content and firmness, showing potential for producing high-quality sweet cherries [88]. Milošević et al. [89] found that rootstocks with stronger growth vigor significantly reduced fruit weight, size, soluble solids content, and titratable acidity, but compared to semi-dwarf and dwarf rootstocks, they increased the content of phenolic compounds.

There is often a significant interaction effect between rootstocks and scions in improving fruit yield and quality. In sweet cherry, it was found that the variety ‘Vera’ (*P. avium*) grafted onto ‘Egervár’ (*P. mahaleb*), ‘Carmen’ (*P. avium*) grafted onto ‘Cemany’ (*P. mahaleb*), and ‘Petrus’ (*P. avium*) grafted onto ‘Magyar’ (*P. mahaleb*) rootstocks achieved the highest yields [90]. For apricot (*P. armeniaca*) varieties ‘E-101’ and ‘E-404’, grafting onto five different rootstocks revealed that the rootstock ‘PAC 00-08’ ((*P. salicina* × *P. cerasifera*) × *P. armeniaca*) significantly increased the firmness of ‘E-101’ fruits, while ‘PADAC 01-47’ ((*P. besseyi* × *P. armeniaca*) × (*P. cerasifera* × *P. armeniaca*)) notably enhanced the firmness of ‘E-404’ fruits [91]. Therefore, research on the function of rootstocks and the interaction between rootstocks and scions is crucial for developing effective breeding strategies.

## 4. Breeding Achievements in Rootstocks of Stone Fruit Trees

**Figure 2:**
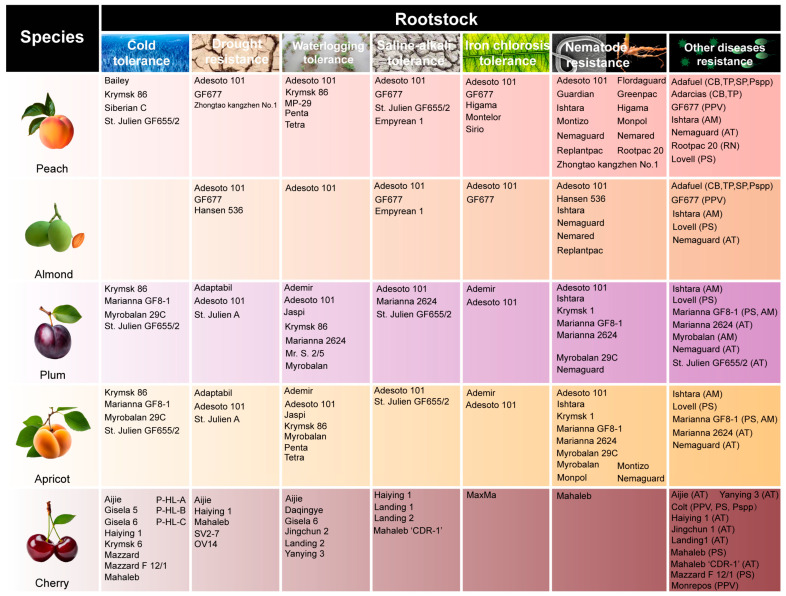
Common resistant rootstocks for stone fruit trees. Note: the abbreviation in parentheses refers to the bacterial species resisted by the rootstock [58,67,92,94,95,96,97,98,99,100]. PPV: *Plum pox virus*; ATs: *Agrobacterium tumefactions*; PS: *Pseudomonas syringae*; AM: *Armillaria mellea*; Pspp: *Phytophthora* spp.; CB: *Corineum beijerinckii*; TP: *Tranzschelia. pruni-spinosae*; SP: *Sphaerotheca pannosa*; RN: *Rosellinia necatrix*.

To date, in pursuit of diverse breeding objectives, breeding scientists have successfully bred a variety of rootstocks for stone fruit trees, each tailored to meet specific needs. For instance, the easily propagated plum rootstock ‘Myrobalan 29C’ was selected from *P. cerasifera* seedlings [92]; the iron chlorosis-tolerant rootstock ‘GF677’ was bred from the hybridization of *P. dulcis* and *P. persica* [34,35]; and the fully dwarfing cherry rootstock ‘Gisela 3’ was developed from the crossbreeding of *P. cerasus* and *P. canescens* [93]. This paper briefly summarizes the main resistance rootstocks (Figure 2) and growth-controlling rootstocks (Table S1) of stone fruit trees [58,67,92,94,95,96,97,98,99,100].

## 5. Molecular Breeding Techniques for Rootstocks of Stone Fruit Trees

Traditional breeding methods include introduction and selection breeding, hybrid breeding, backcross breeding, mutation breeding, distant hybrid breeding, and polyploid breeding. These approaches often have a degree of randomness, and obtaining superior varieties typically requires a long breeding cycle [101]. With the advancement of modern molecular biology, molecular breeding techniques such as marker-assisted selection and genetic engineering have emerged. These technologies allow for the targeted improvement of desired traits, significantly reducing the breeding cycle [102]. Molecular breeding is also increasingly being applied to the breeding of rootstocks for stone fruit trees.

### 5.1. Marker-Assisted Breeding

Marker-assisted breeding is a highly effective method for genetic improvement. By developing molecular markers closely linked to target traits, breeders can predict these traits in varieties at an early stage. This significantly enhances selection efficiency, reduces the randomness in the breeding process, and accelerates the breeding program [103].

In the breeding of rootstocks for stone fruit trees, marker-assisted breeding is mainly used to screen for disease-resistant germplasm. For example, Soriano et al. [104] developed an SSR marker, PGS1.21, which is closely associated with resistance to PPV in apricot and is located within the PPVres locus [105]. In some cases, the molecular assessment of the PGS1.21 marker is not entirely effective, leading to speculation that a second locus associated with PPV resistance may exist [106]. Additionally, an allele variant linked to PPV resistance, ParPMC2, was identified within the PPVres locus [107]. A five base pair deletion in the second exon of this gene is also considered a suitable Indel molecular marker for identifying PPV resistance [108]. However, no reports of molecular marker-assisted evaluation of PPV resistance in Prunus rootstocks have been published to date. Nevertheless, using the developed PPV molecular markers, breeders have identified several PPV-resistant apricot seedlings or cultivars [109,110,111]. It is believed that these materials will become important for future PPV-resistant rootstock breeding programs.

PTSL is a devastating disease in peaches, as it is not easily detectable in the early stages of planting, only becoming apparent after 3 to 5 years, by which time the plants are typically facing death [112,113]. To accurately predict and select superior germplasms resistant to PTSL, Blenda et al. [68] created an F2 population with varying sensitivity to PTSL by crossing the ‘Guardian 3-17-7’ line (PTSL-tolerant) with ‘Nemaguard’ (PTSL-susceptible) and further self-crossing, and using this population along with 151 AFLP and 21 SSR molecular markers, they constructed a genetic linkage map to identify key molecular markers linked to PTSL resistance.

RKN is a major pest causing poor growth in stone fruit trees [114]. Although several peach rootstocks, such as ‘Nemared’, ‘Nemaguard’, and ‘Flordaguard’, have been developed to resist one or more species of RKN, they are not effective against the newly discovered RKN species, *M. floridensis*. To select peach rootstocks with stronger and broader resistance to RKN, breeders have utilized an interspecific hybrid population between ‘Flordaguard’ and *P. kansuensis*, and developed the DP98-025 marker by combining SSR marker analysis, which co-locates with the RKN resistance gene [115]. Duval et al. genotyped and phenotyped individuals of apricot, plum, and peach using established RKN markers, and further developed new resistance-associated molecular markers by analyzing the expression patterns of the RKN resistance gene *RMia* [116,117].

Additionally, molecular markers have been employed to identify rootstock varieties with dwarfing effects. For instance, a dwarfing-related QTL was localized on chromosome 3 of peach through SLAF-seq and genetic analysis, and subsequent development of SNP markers was carried out [118]. It was also found that the peach dwarfing gene *Dw* is located at the distal end of linkage group 6, with the second SNP in its coding sequence co-segregating with the dwarf phenotype, a finding confirmed in the dwarfing peach variety ‘Small Sunning’ [119].

### 5.2. Genetic Engineering Breeding

Plant genetic engineering breeding is an efficient method for obtaining new varieties by utilizing genetic engineering techniques, primarily involving the isolation, modification, chromosomal integration, and in vivo expression of target genes in plants. Currently, due to the lack of natural virus resistance in stone fruits, obtaining stable virus-resistant varieties through genetic modification is a primary focus in the genetic engineering breeding of stone fruit rootstocks. Ten transgenic lines were successfully generated by infecting the hypocotyls of mature European plum seeds with *Agrobacterium rhizogenes*, and grafting experiments confirmed that seven of these lines exhibited resistance to PPV [120]; the plum rootstock ‘Elita’ ((*P. pumila* × *P. salicina*) × *P. cerasifera*) was successfully modified by using RNAi technology, resulting in a high-quality rootstock with resistance to PPV [121]. Similarly, a PPV resistance gene was introduced into the plum rootstock ‘Startovaya’ (*P. domestica*), which remained fully resistant to viral infection for nine years after grafting with scions carrying the PPV virus, yet the transgenic rootstock was unable to provide virus resistance to the scions in this study [122]. Fortunately, recent studies have found that PPV resistance based on RNA silencing can be transferred from transgenic plum rootstocks to non-transgenic apricot scions [123]. Mechanistic studies have demonstrated that PPV translation initiation factors include eIF(iso)4G and eIF(iso)4E, and using RNAi to silence these factors in the sour cherry rootstock ‘146-2’ (*P. pumila* × *P. tomentosa*) showed that silencing eIF(iso)4G does not increase resistance, but silencing eIF(iso)4E enhances resistance to PPV, with no signs of infection observed for two years after inoculation, demonstrating the potential to establish PPV-resistant rootstocks by inhibiting translation initiation factors in clonal rootstocks [124].

In addition to improving rootstock resistance, genetic engineering has also been used to enhance the rooting efficiency of stone fruit rootstocks. For example, after the recombinant gene *PcMPK3-HA* was successfully introduced into the cherry rootstock ‘Gisela 6’, the transgenic lines exhibited a stronger ability to form adventitious roots and positively influenced the root growth of tissue-cultured rootstock seedlings after transplantation [125].

Gene editing, as a method of non-exogenous genome modification, is more widely accepted by the public in terms of food security. Currently, gene editing technology in stone fruit trees is still in the developmental stage. Although recent reports have demonstrated the successful targeting of transcription factors ERF74 and GAI in apricots using a combination of the hairy root transformation system and CRISPR/Cas9 gene editing technology [126], most *Prunus* stone fruit varieties, particularly peaches, still lack stable genetic transformation systems. Establishing efficient genetic transformation methods and overcoming genotype limitations remain the primary tasks in genetic engineering breeding for stone fruit trees. Additionally, even though gene editing does not involve the insertion of foreign genes, the European Court of Justice ruled in 2018 that organisms developed through gene editing technology should still be classified as genetically modified organisms and subjected to the same regulatory obligations [127]. However, some countries around the world tend to adopt stricter regulations for gene-edited organisms, while others may implement more flexible policies. These differences reflect the complexity of the global landscape and the varying stances of nations in scientific, political, and social networks [128]. Therefore, in the current era of rapid biotechnological advancement, the comprehensive application of genetic engineering breeding remains a challenging and long-term process.

## 6. Future Perspectives in Stone Fruit Rootstock Research

**Figure 3:**
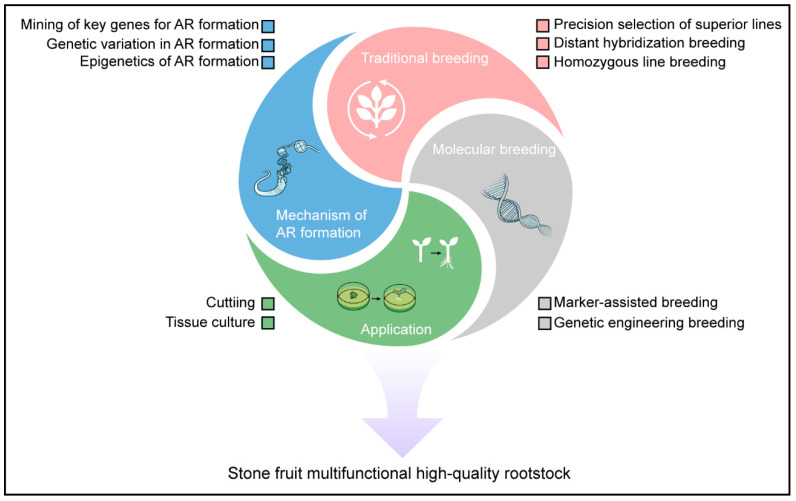
Research directions and breeding strategies for rootstocks of stone fruit trees. AR: adventitious roots.

Modern orchard systems’ success hinges on tailored rootstock solutions—a frontier where apples have set a gold standard. Up to now, breeders have successfully developed a variety of rootstock new varieties with excellent characteristics. However, contemporary horticultural systems face emerging challenges from climate volatility, soil degradation, and evolving pathogen pressures. While biological and economic hurdles persist for stone fruits, emerging biotechnologies and collaborative frameworks offer unprecedented opportunities to develop next-generation rootstocks. This synthesis proposes three strategic research priorities to advance stone fruit rootstock innovation (Figure 3).

### 6.1. Revealing the Molecular Mechanism of Adventitious Root Formation in Rootstocks

Currently, many rootstock germplasm resources of stone fruit trees possess excellent traits that can improve the growth vigor or fruit quality of the scions. However, these rootstocks often have weak rooting abilities and can only be used as interstocks. While interstocks can enhance the dwarfing effect and production efficiency of fruit trees to some extent, they cannot ensure the uniformity of scion traits. Therefore, it is essential to identify the key genes that regulate adventitious root formation in stone fruit tree rootstocks. By investigating the molecular mechanisms of adventitious root initiation from multiple perspectives, including epigenetics and molecular regulation, we can fundamentally address the issue of poor rooting in rootstocks. This will pave the way for gradually replacing interstocks with self-rooted rootstocks.

### 6.2. Developing of Efficient Breeding Strategies and New Techniques for Rootstocks

Although selection breeding and hybrid breeding have relatively low breeding efficiency, they are always basic breeding methods that are technically simple, easy to implement, and effective. In the future, expanding the scale of germplasm screening and the number of hybrid combinations will have practical significance for the cultivation of high-quality rootstocks. Additionally, creating inbred lines through multiple generations of self-fertilization of self-compatible germplasm, and maximizing heterosis through the hybridization of these inbred lines, will also be one of the effective pathways for future rootstock breeding.

Compared to scion cultivar development, stone fruit rootstock breeding programs face significantly extended evaluation cycles, typically requiring 10–15 years or more for commercial validation due to longer juvenile phases and complex trait interactions. These inherent constraints substantially discourage research investment and limit breeder participation in rootstock improvement initiatives. Early molecular marker-assisted breeding has significantly improved breeding efficiency and reduced costs in fruit tree breeding programs. With the evolution of time, biotechnology has been rapidly evolving. The development of novel molecular markers through genome resequencing technology, such as KASP (Kompetitive Allele-Specific PCR), has become a part of modern breeding and will be, or should be, an effective pathway for the future selection and breeding of rootstocks in stone fruit trees. Additionally, elucidating the functions of genes related to important agronomic traits in stone fruit trees and using transgenic or gene-editing technologies to artificially target the creation and improvement of rootstock traits, such as the ease of rooting and dwarfing rootstocks, will also be a focus of future rootstock breeding.

### 6.3. Optimizing Clonal Propagation Techniques for Rootstocks

The clonal propagation of rootstocks is an important factor in ensuring the stable characteristics of scions and achieving high and stable yields. Cutting and tissue culture have long been means of asexual propagation for rootstocks. However, both cutting and tissue culture face genotype-dependent issues, and effective rooting or regeneration methods cannot be widely applied to most high-quality rootstock varieties. Therefore, optimizing the substrate formulations for cuttings and tissue culture and exploring more universal and efficient regeneration and rooting methods will be the direction for the continuous development and improvement of rootstock breeding in stone fruit trees in the future.

Finally, unlike apples’ centralized breeding networks (e.g., Cornell-USDA Geneva program), *Prunus* rootstock research is dispersed across small public institutes (e.g., INRAE France, CRIOF Italy, and many institutions in China) with limited coordination. Therefore, bridging the rootstock innovation gap in stone fruit also demands international collaboration for Prunus germplasm sharing and phenotyping standardization.

## Data Availability

Not applicable.

## Supplementary Materials

The following supporting information can be downloaded at: https://www.mdpi.com/article/10.3390/plants14091320/s1, Table S1: Currently released growth-controlling rootstocks for stone fruit trees.

## References

[1] Riva S.C., Opara U.O., Fawole O.A. (2020). Recent developments on postharvest application of edible coatings on stone fruit: A review. Sci. Hortic..

[2] Food and Agriculture Organization (2022). Food and Agriculture Organization of the United Nations Database (FAOSTAT).

[3] Chen G., Boddu R., Aadil R.M. (2022). Study on Double-Layer Stereo Ecological Cultivation Technology of Greenhouse Gardening Fruit Trees. J. Food Qual..

[4] Ziogas V. (2024). Fruit Growing: Cultivation Strategies for Sustainable Agriculture and Quality Produce. Agronomy.

[5] Torres-Sánchez J., de la Rosa R., León L., Jiménez-Brenes F.M., Kharrat A., López-Granados F. (2021). Quantification of dwarfing effect of different rootstocks in ‘Picual’ olive cultivar using UAV-photogrammetry. Precis. Agric..

[6] Li W., Chu C., Li H., Zhang H., Sun H., Wang S., Wang Z., Li Y., Foster T.M., López-Girona E. (2024). Near-gapless and haplotype-resolved apple genomes provide insights into the genetic basis of rootstock-induced dwarfing. Nat. Genet..

[7] Scalisi A., O’Connell M.G., Stefanelli D., Zhou S., Pitt T., Graetz D., Dodds K., Han L., De Bei R., Stanley J. (2024). Narrow orchard systems for pome and stone fruit—A review. Sci. Hortic..

[8] Liu R., Jia J., Wang C., Wu Q., Du L., Li W., Yang W., Ma J., Zhang D., Xing L. (2025). Transcriptomic and primary metabolic profiles reveal the mechanism of development and maturation of fuji apple grafted onto different dwarfed intermediate rootstocks. Sci. Hortic..

[9] Korkmaz K., Bolat I., Uzun A., Sahin M., Kaya O. (2023). Selection and Molecular Characterization of Promising Plum Rootstocks (*Prunus cerasifera* L.) among Seedling-Origin Trees. Life.

[10] Mayer N.A., Ueno B., Rickes T.B., de Resende M.V.L.A. (2020). Cloning of rootstock selections and *Prunus* spp. cultivars by softwood cuttings. Sci. Hortic..

[11] Justamante M.S., Mhimdi M., Molina-Pérez M., Albacete A., Moreno M.Á., Mataix I., Pérez-Pérez J.M. (2022). Effects of Auxin (Indole-3-butyric Acid) on Adventitious Root Formation in Peach-Based *Prunus* Rootstocks. Plants.

[12] Tsafouros A., Frantzeskaki A., Assimakopoulou A., Roussos P.A. (2019). Spatial and temporal changes of mineral nutrients and carbohydrates in cuttings of four stone fruit rootstocks and their contribution to rooting potential. Sci. Hortic..

[13] Tsafouros A., Roussos P.A. (2020). The possible bottleneck effect of polyamines’ catabolic enzymes in efficient adventitious rooting of two stone fruit rootstocks. J. Plant Physiol..

[14] Blažková J., Hlušičková I. (2002). Testing of wood hardiness to winter freezes in selections from progenies of *Cerapadus* × *Prunus avium* L. crosses. Hortic. Sci..

[15] Turhan E., Ergin S. (2012). Soluble Sugars and Sucrose-Metabolizing Enzymes Related to Cold Acclimation of Sweet Cherry Cultivars Grafted on Different Rootstocks. Sci. World J..

[16] Dogan M., Bolat I., Turan M., Kaya O. (2025). Elucidating stress responses in *Prunus* rootstocks through comprehensive evaluation under drought, heat shock and combined stress conditions. Sci. Hortic..

[17] Cao F., Wei Y., Wang X., Li Y., Peng F. (2019). A Study of the Evaluation of the Pecan Drought Resistance of Grafted ‘Pawnee’ Trees From Different Seedling Rootstocks. HortScience.

[18] Layne R.E.C., Harrison T.B. (1975). ‘Haggith’ Apricot: Rootstock Seed Source. HortScience.

[19] Ljubojević M., Zorić L., Maksimović I., Dulić J., Miodragović M., Barać G., Ognjanov V. (2017). Anatomically assisted cherry rootstock selection. Sci. Hortic..

[20] Jia L.T., Qin X., Lyu D.G., Qin S.J., Zhang P. (2019). ROS production and scavenging in three cherry rootstocks under short-term waterlogging conditions. Sci. Hortic..

[21] Jalili S., Arzani K., Prudencio A.S., Salazar J.A., Martínez-García P.J., Bouzari N., Martínez-Gómez P. (2023). Integrated Morphological, Physiological and Molecular Analysis of the Drought Response in Cultivated and Wild *Prunus* L. Subgenera Cerasus Species. Plant Mol. Biol. Report..

[22] Gerbi H., Paudel I., Zisovich A., Sapir G., Ben-Dor S., Klein T. (2021). Physiological drought resistance mechanisms in wild species vs. rootstocks of almond and plum. Trees.

[23] Martínez-García P.J., Hartung J., Pérez de los Cobos F., Martínez-García P., Jalili S., Sánchez-Roldán J.M., Rubio M., Dicenta F., Martínez-Gómez P. (2020). Temporal Response to Drought Stress in Several *Prunus* Rootstocks and Wild Species. Agronomy.

[24] Jurado-Mañogil C., Martínez-Melgarejo P.A., Martínez-García P., Rubio M., Hernández J.A., Barba-Espín G., Diaz-Vivancos P., Martínez-García P.J. (2024). Comprehensive study of the hormonal, enzymatic and osmoregulatory response to drought in *Prunus* species. Sci. Hortic..

[25] McGee T., Schaffer B., Shahid M.A., Chaparro J.X., Sarkhosh A. (2022). Carbon and nitrogen metabolism in peach trees on different *Prunus* rootstocks in response to flooding. Plant Soil.

[26] Ziegler V.H., Ploschuk E., Weibel A., Insausti P. (2017). Short-term responses to flooding stress of three *Prunus* rootstocks. Sci. Hortic..

[27] Klumb E.K., Braga E.J.B., Bianchi V.J. (2020). Differential expression of genes involved in the response of *Prunus* spp. rootstocks under soil flooding. Sci. Hortic..

[28] Toro G., Pimentel P., Salvatierra A. (2021). Effective Categorization of Tolerance to Salt Stress through Clustering *Prunus* Rootstocks According to Their Physiological Performances. Horticulturae.

[29] Sandhu D., Kaundal A., Acharya B.R., Forest T., Pudussery M.V., Liu X., Ferreira J.F.S., Suarez D.L. (2020). Linking diverse salinity responses of 14 almond rootstocks with physiological, biochemical, and genetic determinants. Sci. Rep..

[30] Shao Y.H., Cheng Y.K., Pang H.G., Chang M.Q., He F., Wang M.M., Davis D.J., Zhang S.X., Betz O., Fleck C. (2021). Investigation of Salt Tolerance Mechanisms Across a Root Developmental Gradient in Almond Rootstocks. Front. Plant Sci..

[31] Paula B.V.d., Marques A.C.R., Rodrigues L.A.T., Souza R.O.S.d., Kulmann M.S.d.S., Kaminski J., Ceretta C.A., Melo G.W.B.d., Mayer N.A., Antunes L.E. (2018). Morphological and kinetic parameters of the uptake of nitrogen forms in clonal peach rootstocks. Sci. Hortic..

[32] Chen Q.J., Lian M., Guo J., Zhang B.B., Yang S.K., Huang K.X., Peng F.T., Xiao Y.S. (2022). Comparative Transcriptome Analysis of Two Peach Rootstocks Uncovers the Effect of Gene Differential Expression on Nitrogen Use Efficiency. Int. J. Mol. Sci..

[33] Aras S., Keles H., Bozkurt E. (2021). Physiological and histological responses of peach plants grafted onto different rootstocks under calcium deficiency conditions. Sci. Hortic..

[34] Sun S.X., Li J., Song H.Y., Chen D., Tu M.Y., Chen Q.Y., Jiang G.L., Zhou Z.Q. (2022). Comparative transcriptome and physiological analyses reveal key factors in the tolerance of peach rootstocks to iron deficiency chlorosis. 3 Biotech.

[35] Jiménez S., Pinochet J., Abadía A., Moreno M.Á., Gogorcena Y. (2008). Tolerance Response to Iron Chlorosis of *Prunus* Selections as Rootstocks. HortScience.

[36] Somavilla L.M., Simão D.G., Tiecher T.L., Hammerschimitt R.K., de Oliveira J.M.S., Mayer N.A., Pavanello E.P., Trentin E., Belles S.W., Brunetto G. (2018). Structural changes in roots of peach rootstock cultivars grown in soil with high zinc content. Sci. Hortic..

[37] Usenik V., Marn M.V. (2016). Sugars and organic acids in plum fruit affected by Plum pox virus. J. Sci. Food Agric..

[38] Zhou J., Xing F., Wang H., Li S. (2021). Occurrence, Distribution, and Genomic Characteristics of Plum Pox Virus Isolates from Common Apricot (*Prunus armeniaca*) and Japanese Apricot (*Prunus mume*) in China. Plant Dis..

[39] Rubio M., Dicenta F., Masse M., Duval H. (2013). Susceptibility of *Prunus* rootstocks against Marcus and Dideron isolates of Plum pox virus by graft-inoculation. Ann. Appl. Biol..

[40] Rubio M., Martínez-Gómez P., Pinochet J., Dicenta F. (2005). Evaluation of resistance to sharka (Plum pox virus) of several *Prunus* rootstocks. Plant Breed..

[41] Tomić J., Glišić I., Milošević N., Štampar F., Mikulič-Petkovšek M., Jakopič J. (2022). Determination of fruit chemical contents of two plum cultivars grafted on four rootstocks. J. Food Compos. Anal..

[42] Dehkordi A., Rubio M., Babaeian N., Albacete A., Martínez-Gómez P. (2018). Phytohormone Signaling of the Resistance to Plum pox virus (PPV, Sharka Disease) Induced by Almond (*Prunus dulcis* (Miller) Webb) Grafting to Peach (*P. persica* L. Batsch). Viruses.

[43] Rubio M., Martínez-García P.J., Martínez-Gómez P., Dicenta F. (2024). Plum pox virus (sharka) resistance in peach by grafting ‘Garrigues’ almond as interstock. Sci. Hortic..

[44] Corell-Sierra J., Corrêa R.L., Gómez G.G., Elena S.F., Oliveros J.C., Rodamilans B., Martínez-García P.J., Martínez-Gómez P., Rubio M. (2024). Almond Grafting for Plum Pox Virus Resistance Triggers Significant Transcriptomic and Epigenetic Shifts in Peaches. Int. J. Mol. Sci..

[45] Liang C., Wan T., Wu R., Zhao M., Zhao Y., Cai Y. (2020). Resistance analysis of cherry rootstock ‘CDR-1’ (*Prunus mahaleb*) to crown gall disease. BMC Plant Biol..

[46] Thomidis T., Exadaktylou E., Tsipouridis C. (2005). Susceptibility of five *Prunus* rootstocks to Agrobacterium tumefaciens. N. Z. J. Crop Hortic. Sci..

[47] Greco D., Aprile A., De Bellis L., Luvisi A. (2021). Diseases Caused by Xylella fastidiosa in *Prunus* Genus: An Overview of the Research on an Increasingly Widespread Pathogen. Front. Plant Sci..

[48] Matsumoto G.O., Febres V.J., Harmon P.F., Chaparro J.X. (2023). Survey of Xylella fastidiosa Infection in *Prunus* Germplasm in Gainesville, FL, USA. HortScience.

[49] Rapicavoli J., Ingel B., Blanco-Ulate B., Cantu D., Roper C. (2017). Xylella fastidiosa: An examination of a re-emerging plant pathogen. Mol. Plant Pathol..

[50] Moll L., Giralt N., Planas M., Feliu L., Montesinos E., Bonaterra A., Badosa E. (2024). *Prunus dulcis* response to novel defense elicitor peptides and control of Xylella fastidiosa infections. Plant Cell Rep..

[51] Beckman T.G. (2008). ‘Sharpe’, a Clonal Plum Rootstock for Peach. HortScience.

[52] Beckman T.G. (2012). ‘MP-29’, a Clonal Interspecific Hybrid Rootstock for Peach. HortScience.

[53] Baumgartner K., Fujiyoshi P., Ledbetter C., Duncan R., Kluepfel D.A. (2018). Screening Almond Rootstocks for Sources of Resistance to Armillaria Root Disease. HortScience.

[54] Beluzán F., Armengol J., Abad-Campos P. (2023). Pathogenicity of Oomycete Species to Different *Prunus* Hybrid Rootstocks. Plant Dis..

[55] Türkölmez Ş., Derviş S., Çiftçi O., Ulubaş Serçe Ç. (2016). First Report of Phytophthora chlamydospora Causing Root and Crown Rot on Almond (*Prunus dulcis*) Trees in Turkey. Plant Dis..

[56] Browne G.T., Ott N.J., Forbes H., Yaghmour M.A., Milliron L.K. (2020). First Report of Phytophthora chlamydospora Causing Crown and Root Rot on Almond in California. Plant Dis..

[57] Browne G.T. (2017). Resistance to Phytophthora Species among Rootstocks for Cultivated *Prunus* Species. HortScience.

[58] Lesmes-Vesga R.A., Cano L.M., Ritenour M.A., Sarkhosh A., Chaparro J.X., Rossi L. (2022). Rootstocks for Commercial Peach Production in the Southeastern United States: Current Research, Challenges, and Opportunities. Horticulturae.

[59] Eliwa G.I., Hagag E.S. (2021). Approach to New peach rootstocks resistant to root-knot nematodes (*Meloidogyne* species) selected from local Mit-Ghamer peach cultivar. Sci. Hortic..

[60] Rubio-Cabetas M.J., Lecouls A.C., Salesses G., Bonnet A., Esmenjaud D. (1998). Evidence of a new gene for high resistance to *Meloidogyne* spp. in *Myrobalan plum*, *Prunus cerasifera*. Plant Breed..

[61] Lecouls A.C., Rubio-Cabetas M.J., Minot J.C., Voisin R., Bonnet A., Salesses G., Dirlewanger E., Esmenjaud D. (1999). RAPD and SCAR markers linked to the Ma1root-knot nematode resistance gene in *Myrobalan plum* (*Prunus cerasifera* Ehr.). Theor. Appl. Genet..

[62] Liu J., Zhu J., Li H., Luo D., Xie J., Li H., Liu S., Zhang Y., Chen L., Xie X. (2023). A preliminary study on the root-knot nematode resistance of a cherry plum cultivar Mirabolano 29C. Czech J. Genet. Plant Breed..

[63] Ji P., Liang C., Yang Y., Wang R., Wang Y., Yuan M., Qiu Z., Cheng Y., Liu J., Li D. (2022). Comparisons of Anatomical Characteristics and Transcriptomic Differences between Heterografts and Homografts in *Pyrus* L.. Plants.

[64] Reig G., Salazar A., Zarrouk O., Forcada C.F.I., Val J., Moreno M.Á. (2019). Long-term graft compatibility study of peach-almond hybrid and plum based rootstocks budded with European and Japanese plums. Sci. Hortic..

[65] Irisarri P., Errea P., Pina A. (2021). Physiological and Molecular Characterization of New Apricot Cultivars Grafted on Different *Prunus* Rootstocks. Agronomy.

[66] Reig G., Zarrouk O., Font i Forcada C., Moreno M.Á. (2018). Anatomical graft compatibility study between apricot cultivars and different plum based rootstocks. Sci. Hortic..

[67] Mendelné Pászti E., Bujdoso G., Ercisli S., Hrotkó K., Mendel Á. (2023). Apricot Rootstocks with Potential in Hungary. Horticulturae.

[68] Bouhadida M., Casas A.M., Gonzalo M.J., Arús P., Moreno M.Á., Gogorcena Y. (2009). Molecular characterization and genetic diversity of *Prunus* rootstocks. Sci. Hortic..

[69] Ben Yahmed J., Ghrab M., Benmoussa H., Ben Mimoun M. (2022). Physiological behavior and nutritional status of almond scion-rootstock combinations in a high-density planting system under warm Mediterranean conditions. Sci. Hortic..

[70] Mestre L., Reig G., Betrán J.A., Pinochet J., Moreno M.Á. (2015). Influence of peach–almond hybrids and plum-based rootstocks on mineral nutrition and yield characteristics of ‘Big Top’ nectarine in replant and heavy-calcareous soil conditions. Sci. Hortic..

[71] Reig G., Mestre L., Betrán J.A., Pinochet J., Moreno M.Á. (2016). Agronomic and physicochemical fruit properties of ‘Big Top’ nectarine budded on peach and plum based rootstocks in Mediterranean conditions. Sci. Hortic..

[72] Reig G., Garanto X., Mas N., Iglesias I. (2020). Long-term agronomical performance and iron chlorosis susceptibility of several *Prunus* rootstocks grown under loamy and calcareous soil conditions. Sci. Hortic..

[73] Reig G., Iglesias I., Zazurca L., Torguet L., Martinez G., Miarnau X. (2022). Physiological and Agronomical Responses of ‘Vairo’ Almond and ‘Big Top’ Nectarine Cultivars Grafted onto Different *Prunus* Rootstocks and Grown under Semiarid Mediterranean Conditions. Agronomy.

[74] Montesinos Á., Rubio-Cabetas M.J., Grimplet J. (2023). Characterization of Almond Scion/Rootstock Communication in Cultivar and Rootstock Tissues through an RNA-Seq Approach. Plants.

[75] Liu Q., Zhu D., Wang J., Zhang L., Hong P., Gong Q. (2023). The evaluation of sweet cherry rootstocks and their application prospects in the world. Deciduous Fruits.

[76] Skočajić D., Gašić U., Dabić Zagorac D., Nešić M., Tešić Ž., Meland M., Fotirić Akšić M. (2021). Analysis of Phenolic Compounds for the Determination of Grafts (in) Compatibility Using In Vitro Callus Cultures of Sato-Zakura Cherries. Plants.

[77] Jalali A., Moghaddam E.G., Marjani A. (2024). Early detection of graft incompatibility in sweet cherry by internode association and callus fusion techniques. Plant Cell Tissue Organ Cult. (PCTOC).

[78] Iglesias I., Botet R. (2024). The selection of appropriate rootstock and training system towards sustainable production of stone fruits. Italus Hortus.

[79] Clark J.R., Finn C.E. (2006). Register of New Fruit and Nut Cultivars List 43. HortScience.

[80] Lordan J., Zazurca L., Maldonado M., Torguet L., Alegre S., Miarnau X. (2019). Horticultural performance of ‘Marinada’ and ‘Vairo’ almond cultivars grown on a genetically diverse set of rootstocks. Sci. Hortic..

[81] Cheng J., Zhang M., Tan B., Jiang Y., Zheng X., Ye X., Guo Z., Xiong T., Wang W., Li J. (2019). A single nucleotide mutation in GID1c disrupts its interaction with DELLA1 and causes a GA-insensitive dwarf phenotype in peach. Plant Biotechnol. J..

[82] Khadivi-Khub A., Anjam K. (2016). *Prunus* scoparia, a suitable rootstock for almond (*Prunus dulcis*) under drought condition based on vegetative and fruit characteristics. Sci. Hortic..

[83] Reig G., Font i Forcada C., Mestre L., Betrán J.A., Moreno M.Á. (2018). Potential of new *Prunus cerasifera* based rootstocks for adapting under heavy and calcareous soil conditions. Sci. Hortic..

[84] Yaman M., Uğur R., Sümbül A., Keçe Y., Gönültaş M., Ünsal H.T., Güneş A., Yildiz E., Yilmaz K.U. (2024). Determination of fruit characteristics, nutrients and biochemical contents of Transvalia (*Prunus persica* L.) peach cultivar grafted on different clonal rootstocks obtained by selection and hybridization. Sci. Hortic..

[85] Font i Forcada C., Gogorcena Y., Moreno M.A. (2013). Fruit sugar profile and antioxidants of peach and nectarine cultivars on almond×peach hybrid rootstocks. Sci. Hortic..

[86] Giorgi M., Capocasa F., Scalzo J., Murri G., Battino M., Mezzetti B. (2005). The rootstock effects on plant adaptability, production, fruit quality, and nutrition in the peach (cv. ‘Suncrest’). Sci. Hortic..

[87] Iglesias I., Giné-Bordonaba J., Garanto X., Reig G. (2019). Rootstock affects quality and phytochemical composition of ‘Big Top’ nectarine fruits grown under hot climatic conditions. Sci. Hortic..

[88] López-Ortega G., García-Montiel F., Bayo-Canha A., Frutos-Ruiz C., Frutos-Tomás D. (2016). Rootstock effects on the growth, yield and fruit quality of sweet cherry cv. ‘Newstar’ in the growing conditions of the Region of Murcia. Sci. Hortic..

[89] Milošević T., Milošević N., Mladenović J. (2020). Combining fruit quality and main antioxidant attributes in the sour cherry: The role of new clonal rootstock. Sci. Hortic..

[90] Bujdosó G., Magyar L., Hrotkó K. (2019). Long term evaluation of growth and cropping of sweet cherry (*Prunus avium* L.) varieties on different rootstocks under Hungarian soil and climatic conditions. Sci. Hortic..

[91] Hernández F., Pinochet J., Moreno M.A., Martínez J.J., Legua P. (2010). Performance of *Prunus* rootstocks for apricot in Mediterranean conditions. Sci. Hortic..

[92] Kumar A., Rathore J., Waida U.I., Sharma A., Nagar P., Mir M. (2021). Rootstocks of Stone Fruit Crops. Production Technology of Stone Fruits.

[93] Gruppe W. (1985). An overview of the cherry rootstock breeding programme. Acta Hortic..

[94] Zhu D., Wang J., Hong P., Tan Y., Liu Q. (2020). A new tetraploid sweet cherry dwarf rootstock cultivar ‘Aijie’. Acta Hortic. Sin..

[95] Liu L., Zhang J., Dun B., Li M., Li C. (2014). Breeding of a new sweet cherry rootstock cultivar ‘Haiying 1’. J. Fruit Sci..

[96] Zhang X., Yan G., Zhou Y., Wang J., Duan X., Wu C., Zhang K. (2022). A new sweet cherry rootstock cultivar ‘Jingchun 2’. Acta Hortic. Sin..

[97] Zhang X., Yan G., Zhou Y., Wang J., Duan X., Zhang K. (2021). A new sweet cherry rootstock cultivar ‘Jingchun 1’. Acta Hortic. Sin..

[98] Zhang X., Yan G., Zhou Y., Wang J., Duan X., Zhang K. (2021). A new sweet cherry rootstock cultivar ‘Landing 3’. Acta Hortic. Sin..

[99] Zhang X., Zhang F.X., Sun Q.T., Li Y.J., Tian C.P., Li F.D., Wang Y.X., Li S.P. (2018). Selection and cultivation of sweet cherry rootstock ‘Yanying No. 3’. Yantai Fruits.

[100] Wang L.-R., Wang X.-W., Zhu G.-R., Fang W.-C., Chen C.-W., Cao K., Li Y., Wu J.-L., Wang L.-L., Niu P. (2023). A replantation disease resistant new peach rootstock cultivar Zhong Tao Kang Zhen No.1. J. Fruit Sci..

[101] Bowman K.D., McCollum G., Albrecht U. (2021). SuperSour: A New Strategy for Breeding Superior Citrus Rootstocks. Front. Plant Sci..

[102] Vahdati K., Sarikhani S., Arab M.M., Leslie C.A., Dandekar A.M., Aletà N., Bielsa B., Gradziel T.M., Montesinos Á., Rubio-Cabetas M.J. (2021). Advances in Rootstock Breeding of Nut Trees: Objectives and Strategies. Plants.

[103] Lambert P., Campoy J.A., Pacheco I., Mauroux J.-B., Da Silva Linge C., Micheletti D., Bassi D., Rossini L., Dirlewanger E., Pascal T. (2016). Identifying SNP markers tightly associated with six major genes in peach [*Prunus persica* (L.) Batsch] using a high-density SNP array with an objective of marker-assisted selection (MAS). Tree Genet. Genomes.

[104] Soriano J.M., Domingo M.L., Zuriaga E., Romero C., Zhebentyayeva T., Abbott A.G., Badenes M.L. (2011). Identification of simple sequence repeat markers tightly linked to plum pox virus resistance in apricot. Mol. Breed..

[105] Zuriaga E., Soriano J.M., Zhebentyayeva T., Romero C., Dardick C., Cañizares J., Badenes M.L. (2013). Genomic analysis reveals MATH gene(s) as candidate(s) for Plum pox virus (PPV) resistance in apricot (*Prunus armeniaca* L.). Mol. Plant Pathol..

[106] Rubio M., Ruiz D., Egea J., Martínez-Gómez P., Dicenta F. (2014). Opportunities of marker-assisted selection for Plum pox virus resistance in apricot breeding programs. Tree Genet. Genomes.

[107] Zuriaga E., Romero C., Blanca J.M., Badenes M.L. (2018). Resistance to Plum Pox Virus (PPV) in apricot (*Prunus armeniaca* L.) is associated with down-regulation of two MATHd genes. BMC Plant Biol..

[108] Polo-Oltra Á., Romero C., López I., Badenes M., Zuriaga E. (2020). Cost-Effective and Time-Efficient Molecular Assisted Selection for PPV Resistance in Apricot Based on ParPMC2 Allele-Specific PCR. Agronomy.

[109] Gürcan K., Çetinsağ N., Pınar H., Macit T. (2019). Molecular and biological assessment reveals sources of resistance to Plum pox virus—Turkey strain in Turkish apricot (*Prunus armeniaca*) germplasm. Sci. Hortic..

[110] Rubio M., Martínez-Gómez P., Dicenta F. (2023). Apricot breeding for multiple resistance to Plum pox virus and Apple chlorotic leaf spot virus. Sci. Hortic..

[111] Nicolás-Almansa M., Ruiz D., Salazar J.A., Guevara A., Cos J., Martínez-Gómez P., Rubio M. (2023). Phenotypic and molecular characterization of new interspecific Japanese plum × apricot hybrids (plumcots). Sci. Hortic..

[112] Mayer N.A., Ueno B., Nava G., Bianchi V.J., Nicolao G., Roth F.M., Antunes L.E.C. (2024). Performance of clonal rootstocks for peach and own-rooted ‘Maciel’ trees in area with a history of the PTSL syndrome. Sci. Hortic..

[113] Blenda A.V., Verde I., Georgi L.L., Reighard G.L., Forrest S.D., Muñoz-Torres M., Baird W.V., Abbott A.G. (2007). Construction of a genetic linkage map and identification of molecular markers in peach rootstocks for response to peach tree short life syndrome. Tree Genet. Genomes.

[114] Meza P., Soto B., Rojas L., Esmenjaud D. (2016). Identification of *Meloidogyne* Species from the Central Valley of Chile and Interaction with Stone Fruit Rootstocks. Plant Dis..

[115] Maquilan M.A.D., Olmstead M.A., Olmstead J.W., Dickson D.W., Chaparro J.X. (2018). Genetic analyses of resistance to the peach root-knot nematode (*Meloidogyne floridensis*) using microsatellite markers. Tree Genet. Genomes.

[116] Duval H., Heurtevin L., Dlalah N., Caravel C., Callot C., Van Ghelder C. (2024). Identification and Expression of the peach TNL RMia genes for the Resistance to the Root-knot Nematode *Meloidogyne incognita*. bioRxiv.

[117] Duval H., Van Ghelder C., Portier U., Confolent C., Meza P., Esmenjaud D. (2019). New Data Completing the Spectrum of theMa, RMia, andRMjaGenes for Resistance to Root-Knot Nematodes (*Meloidogyne* spp.) in *Prunus*. Phytopathology.

[118] Lu Z., Niu L., Chagné D., Cui G., Pan L., Foster T., Zhang R., Zeng W., Wang Z. (2016). Fine mapping of the temperature-sensitive semi-dwarf (Tssd) locus regulating the internode length in peach (*Prunus persica*). Mol. Breed..

[119] Cantín C.M., Arús P., Eduardo I. (2018). Identification of a new allele of the Dw gene causing brachytic dwarfing in peach. BMC Res. Notes.

[120] García-Almodóvar R.C., Clemente-Moreno M.J., Díaz-Vivancos P., Petri C., Rubio M., Padilla I.M.G., Ilardi V., Burgos L. (2014). Greenhouse evaluation confirms in vitro sharka resistance of genetically engineered h-UTR/P1 plum plants. Plant Cell Tissue Organ Cult. (PCTOC).

[121] Sidorova T., Pushin A., Miroshnichenko D., Dolgov S. (2017). Generation of Transgenic Rootstock Plum ((*Prunus pumila* L. × *P. salicina* Lindl.) × (*P. cerasifera* Ehrh.)) Using Hairpin-RNA Construct for Resistance to the Plum pox virus. Agronomy.

[122] Sidorova T., Mikhailov R., Pushin A., Miroshnichenko D., Dolgov S. (2019). Agrobacterium-Mediated Transformation of Russian Commercial Plum cv. “Startovaya” (*Prunus domestica* L.) With Virus-Derived Hairpin RNA Construct Confers Durable Resistance to PPV Infection in Mature Plants. Front. Plant Sci..

[123] Alburquerque N., Pérez-Caselles C., Faize L., Ilardi V., Burgos L. (2023). Trans-grafting plum pox virus resistance from transgenic plum rootstocks to apricot scions. Front. Plant Sci..

[124] Mourenets L., Pushin A., Timerbaev V., Khmelnitskaya T., Gribkov E., Andreev N., Dolgov S. (2022). Effect of Gene Silencing of Translation Initiation Factors eIF(iso)4G and eIF(iso)4E on Sour Cherry Rootstock Resistance to Sharka Disease. Int. J. Mol. Sci..

[125] Zong X.J., Xu L., Tan Y., Wei H.R. (2022). Development of genetically modified sweet cherry rootstock ‘Gisela 6’ with overexpression of PcMPK3-HA gene by Agrobacterium-mediated genetic transformation. Plant Cell Tissue Organ Cult..

[126] Jedličková V., Štefková M., Sánchez López J.F., Grimplet J., Rubio Cabetas M.J., Robert H.S. (2024). Genome editing in almond using hairy root transformation system. Plant Cell Tissue Organ Cult. (PCTOC).

[127] Wasmer M. (2019). Roads Forward for European GMO Policy—Uncertainties in Wake of ECJ Judgment Have to be Mitigated by Regulatory Reform. Front. Bioeng. Biotechnol..

[128] Escajedo San-Epifanio L., Filibi I., Lasa López A., Puigdomènech P., Uncetabarrenechea Larrabe J. (2023). Possible EU futures for CRISPR-edited plants: Little margin for optimism?. Front. Plant Sci..

